# Metastatic Ectopic Thymoma of the Neck without Mediastinal Involvement

**DOI:** 10.22038/IJORL.2022.60775.3097

**Published:** 2022-09

**Authors:** Jesu Susan Jose, Nidhi Gupta, Awadhesh K Pandey, Kislay Dimri, Nitin Gupta

**Affiliations:** 1 *Department of Radiation Oncology, Government Medical College and Hospital Chandigarh, Chandigarh, India.*; 2 *Department of ENT & Head and Neck Surgery, Government Medical College and Hospital Chandigarh, Chandigarh, India.*

**Keywords:** Diagnosis, Ectopic, Lung metastases, Management, Neck, Thymoma.

## Abstract

**Introduction:**

Thymoma is a rare malignancy with usual location in the antero-superior mediastinum. Occurrence of an extra-mediastinal thymic malignancy in the neck with lung metastasis and without involvement of the mediastinum is an extremely rare condition. Staging systems and treatment guidelines are defined for mediastinal thymomas but not for ectopically located thymomas.

**Case Report:**

38-year-old female presented with the chief complaint of a progressive neck swelling, located predominantly in the right lateral neck, extending to the midline. Computed Tomography showed a heterogenous peripherally enhancing mass with likely origin from the thyroid gland. The mass measured 12 x 6 x 3.5 cm in size and extended from the hyoid bone superiorly to the suprasternal location inferiorly. Additionally, there were multiple, variable sized subpleural nodules scattered in both lungs, suggestive of lung metastases. Histopathology and immunohistochemistry findings from neck mass confirmed the diagnosis of Thymoma Type A.

**Conclusions:**

Thymoma is a rare tumor that typically does not show aggressive behaviour. Extra-mediastinal neck thymoma with bilateral lung metastasis is an extremely rare presentation. Thymoma presenting as neck swelling without mediastinal extension on radiology, poses a diagnostic dilemma. Histopathology with immunohistochemistry helps to confirm the final diagnosis. Surgery is the mainstay for the management of localized tumors with adjuvant treatment reserved for incompletely resected tumors or advanced stage. Systemic metastasis are rare in this indolent tumor and chemotherapy regimens are investigational. Clinical presentation, prognostic factors, staging and management guidelines are still not well defined for this rare tumor with atypical location.

## Introduction

Thymoma is a rare neoplasm arising from thymic epithelial cells, 90% of which are present within the anterior mediastinum and the rest occur in the middle and posterior mediastinum (1). 

These rare tumors usually present in the 4th decade and are commonly associated with myasthenia gravis. In patients without myasthenia gravis, the peak age of presentation is in the seventh decade or later (2-7). The incidence of thymoma reported by Surveillance Epidemiology and End Results (SEER) analysis is around 0.13 per 100,000 person-years (8). Ectopic thymoma is an even rare entity constituting approximately 4% of the thymomas (9-11). 

Embryologically, thymus has an endodermal origin from the lower part of the third pharyngeal pouch which originates in the neck during the early foetal life and reaches the mediastinum after descend. Rarely, the thymus fails to descend and may appear as a remnant along the cervical pathway from the angle of mandible to the thyroid gland; thyroid gland being the most common site of ectopic thymic tissue. 

Ectopic thymus has been most commonly reported in prepubertal paediatric populations due to age-related involution. These ectopic thymic tissues may develop hyperplasia or thymic neoplasms like thymoma, thymic carcinoma and lymphomas (12-14). Thymomas usually have indolent growth but can be locally invasive. 

## Case Report

**Fig 1:**
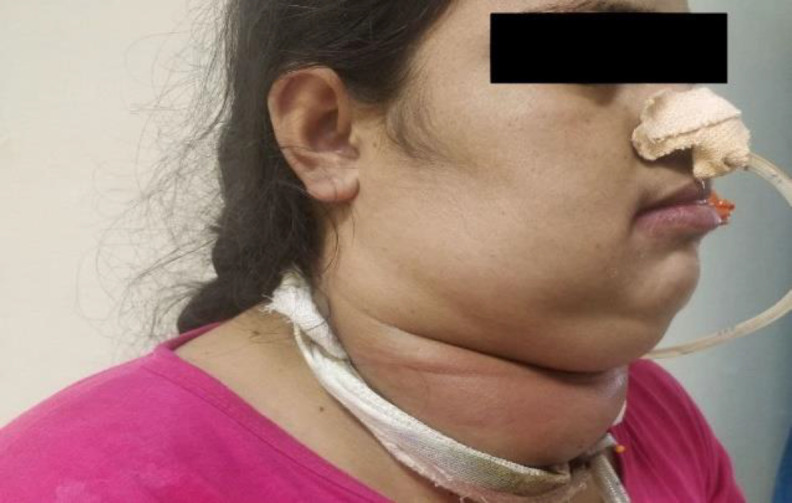
Picture of 38-year-old female with diffuse neck swelling occupying most of anterior aspect of neck

*A 38-year-old premenopausal woman presented to us with the chief complaint of a progressive neck swelling which appeared 8 months ago and was initially located in the right side of the neck but gradually progressed to involve the anterior neck also (* Fig.1 *). The swelling was associated with difficulty in breathing and voice change. On physical examination, there was a visible neck mass occupying the anterior and right lateral side of neck, measuring approximately* *12 x 6 x 3.5 cm in size* *. The swelling had a smooth surface, was firm in consistency, non-mobile, non-tender, extending superiorly from the level of hyoid bone to the suprasternal notch inferiorly. Laterally the swelling extended from right sternocleidomastoid muscle to the left sternocleidomastoid muscle. The swelling did not move with tongue protrusion or deglutition. On examination, Kocher’s sign and Pemberton sign were absent. On palpation using Lahey’s method, separate lobes of the swelling could not be appreciated. Mobility in both vertical and horizontal planes was restricted. No signs of thyrotoxicosis were seen.*

*All blood investigations including hemogram, peripheral blood film, renal function tests, liver function tests and serum electrolytes were within normal range. Thyroid function tests (TFT) showed Free triiodothyronine value of 2.17pg/ml (low) and Thyroid Stimulating Hormone (TSH) value of 34.03 (high) suggestive of hypothyroidism. Serum alpha-feto protein (AFP) and beta-human chorionic gonadotrophin (HCG) were within normal limits. Anti-acetyl choline receptor antibodies, diagnostic of myasthenia gravis, were not detected. Ophthalmic examination revealed restriction of lateral extraocular movements in both eyes. Seventy-degree endoscopic examination of larynx revealed right side vocal cord palsy.*

**Fig 2:**
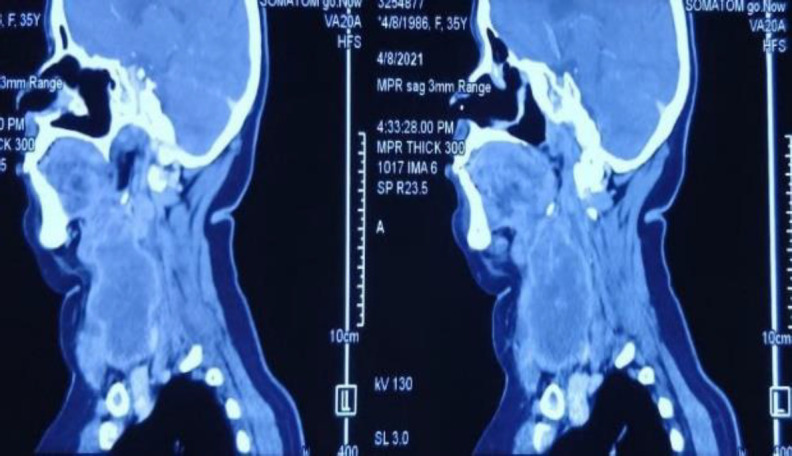
Contrast CT of neck showing cranio-caudal extent of the neck mass starting superiorly from hyoid to D1 vertebra with no retrosternal extension

**Fig 3:**
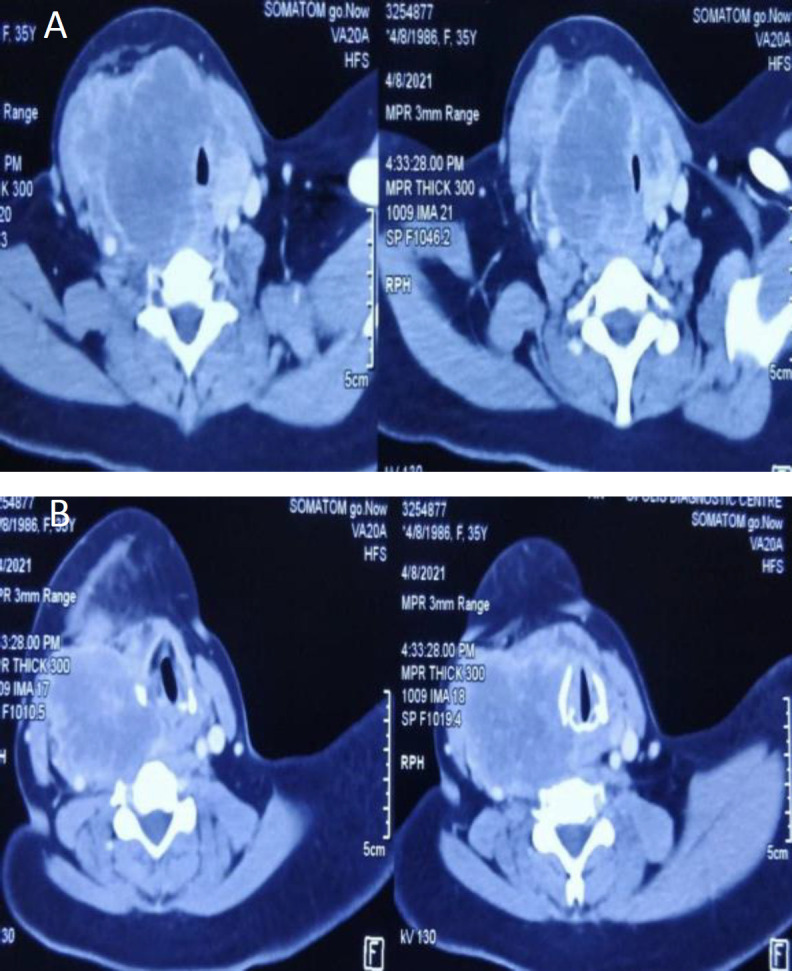
Axial section across neck showing peripherally enhancing mass visualized on right lateral side of neck not separable from right thyroid lobe; involving sternocleidomastoid muscle on the right side and compressing trachea as seen in Figure 3A and 3B

*Contrast-enhanced computed tomography (CECT) of the neck and chest showed a large heterogenous peripherally enhancing mass measuring 5.9 x 7.8 x 7.9 cm predominantly on the right side of neck likely arising from the right lobe of thyroid gland. The mass also involved the left lobe of thyroid and extended from the hyoid level superiorly to thoracic D1 vertebra inferiorly. No retrosternal extension was seen. Superiorly, the mass was abutting the submandibular gland with ill-defined fat planes. Medially, it was compressing the larynx, trachea and the oesophagus without involvement of the laryngeal cartilages. Laterally, it involved the sternocleidomastoid muscle on the right side. Posteriorly, it extended from C3 to C7 levels. A prominent lymph node 13 x 9 mm size was seen at level II on the right side (* Fig.2 *,* Fig.3A, Fig. 3B *). CECT Chest showed multiple, variable-sized, subpleural and parenchymal nodules, diffusely scattered in both lungs with the largest in the left lower lobe, 12 x 12 mm in size. CT orbit and MRI brain were normal.*

**Fig 4:**
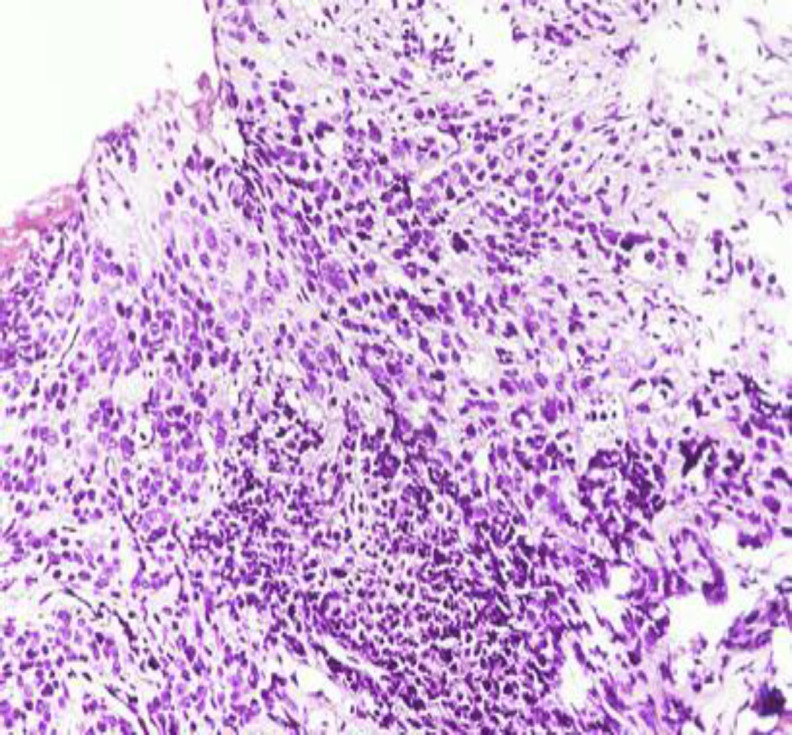
Hematoxylin and eosin stain shows oval shaped epithelial cells arranged in nests and islands with brisk mitoses

*The patient underwent an incisional biopsy from the neck swelling and an emergency tracheostomy for stridor. The histopathological report revealed thymoma Type A (atypical) with epithelial cells arranged in nests and islands (* Fig.4 *).*

**Fig 5:**
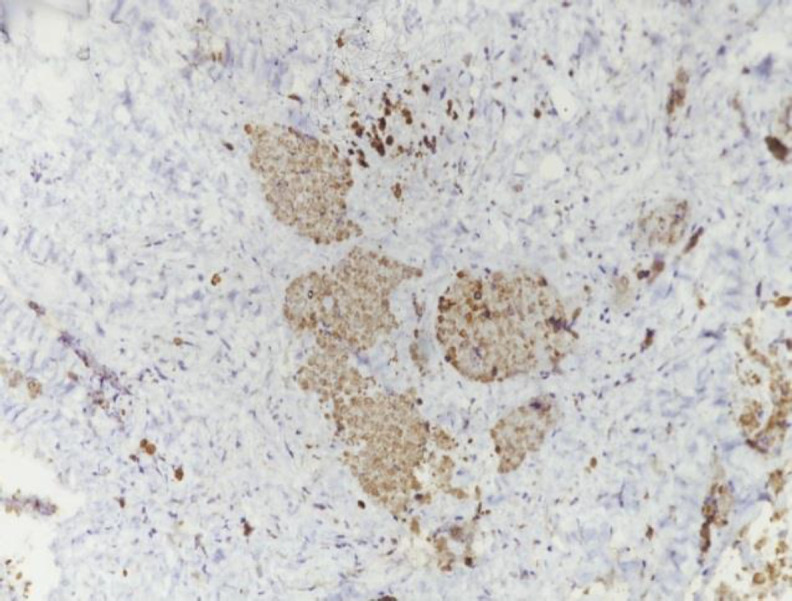
Immunohistochemistry showed tumor cells to be positive for cytokeratin

**Fig 6:**
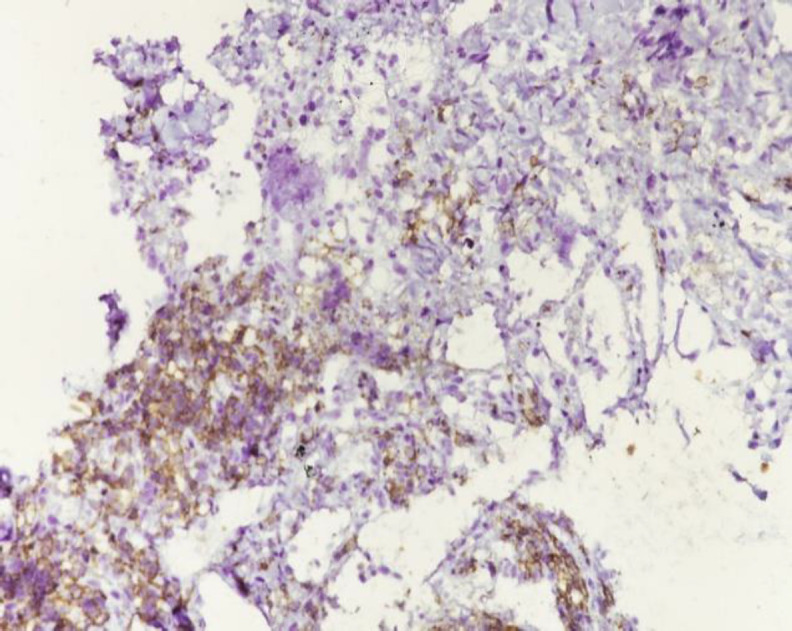
Membranous staining for leucocyte common antigen

*The immunohistochemistry showed strong positivity for cytokeratin and leucocyte common antigen (* Fig.5 *,* Fig.6 *). The patient was treated with palliative intent in view of multiple pulmonary metastases. She was planned for palliative chemotherapy with CAP regimen consisting of Inj.Cisplatin 50mg/m2, Inj.Doxorubicin 50mg/m2 and Inj. Cyclophosphamide 500mg/m2. She received 2 cycles of chemotherapy till her last follow up.*

## Discussion

The thymus gland is a lymphoepithelial organ, located in the anterior mediastinum which helps in the production and maturation of immune cells (15). Thymomas most commonly present as a mediastinal mass with extension to the neck. Ectopic presentation of the thymoma in the neck without any mediastinal involvement along with lung metastasis is extremely rare. Our patient was evaluated for suspicion of thyroid malignancy based on the location of neck mass. CT scan of the neck revealed a heterogeneously enhancing mass, likely arising from the right lobe of the thyroid gland. The mass was extending from hyoid to D1 vertebrae without any retrosternal extension. 

Chan et al have reported 16 cases of ectopic cervical thymomas. In their series, thymomas were more commonly seen in females of middle age and were commonly left-sided in location (16). All these characteristics matched with our patient. Cervical thymomas are usually indolent but our patient’s history revealed an aggressive course with a rapid onset over 8 months. The patient presented with a bulky neck mass requiring urgent tracheostomy and had extensive lung metastasis.

Clinical symptoms for mediastinal tumors are more specific and include breathlessness, chest pain, cough etc. However, for patients with cervical thymoma, symptoms are non-specific and are usually associated with mass effect on neck structures like pain, earache, dysphagia, hoarseness and dyspnoea in advanced cases. These non-specific symptoms further lead to a variety of differential diagnosis. Myasthenia gravis commonly associated with mediastinal thymomas facilitates early diagnosis of mediastinal thymoma while cervical thymomas are rarely associated with myasthenia gravis further delaying definitive diagnosis. Our patient presented with progressive neck swelling, which was not associated with myasthenia gravis and anti-acetyl choline receptor antibodies were negative.

Tissue diagnosis with immunohistochemistry remains the key to diagnosis and cytology is not very useful. Thymoma is a neoplasm of thymic epithelial tumors with a variable component of lymphoid tissue. Many other tumors like lymphomas and germ cell tumors can arise within the thymus but thymomas and thymic carcinomas arise only from the true thymus elements (15). On histopathology, thymoma includes epidermoid cells, squamous cells, spindle cells, papillary lesions and degenerated Hassall’s corpuscles. Thymic lymphomas present with limited or generalized enlargement of thymus gland by lymphocytic proliferation and the replacement of normal architecture of thymus by a population of lymphoblasts (17). The histopathological picture in our patient showed a tumor comprising of epithelial components arranged in nests with moderate anaplasia. The tumor cells were positive for cytokeratin with few interspersed lymphoid cells. Immunocytochemical staining with cytokeratin shows the epithelial nature of the neoplastic cells (18). Leucocyte common antigen (LCA) has been used to target lymphocytic cells of thymoma which usually consist of immature T-cells, although sometimes a small number of CD-20 positive B lymphocytes can also be found. Histochemical staining with LCA shows the presence of lymphoid components of thymoma. Cytokeratin, Pax8, CD1a, CD99 and LCA can be present in thymomas while cytokeratin and Pax8 are usually absent in T-cell lymphoblastic lymphoma (19). Other commonly used markers for T-lymphocytes include CD3, CD5, Terminal deoxynucleotidyl transferase (Tdt) and Ki67. Studies also suggest a high correlation between CD57 positivity and neuromuscular disease (18). Some of the commonly used thyroid cell specific markers include thyroglobulin, thyroid transcription factor-1, thioperoxide and dipeptidyl aminopeptidase-4. Neuroendocrine markers like calcitonin, parathyroid hormone and chromogranin can be used to rule out cells of thyroid or parathyroid origin. The histological absence of thyroid follicles and colloid, point to the absence of thyroid tissue component in the biopsy. 

The histopathological and histochemical features seen in our patient are consistent with neoplastic thymic epithelial cells with few lymphocytes.

The histological classification gives an insight into the malignant potential of the tumor and the most common histological presentation is WHO Type A. The tumor in our patient was reported as atypical variant of WHO Type A on histopathology. Type A variant consists of spindle-shaped epithelial cells with paucity or absence of immature (TdT+) T cells throughout the tumor. In addition, the atypical variant of Type A thymoma includes comedo-type tumor necrosis, increased mitotic count and nuclear crowding. It is also called as spindle cell thymoma or medullary thymoma and usually has a good prognosis (20). Biochemical markers like AFP, Beta- HCG help to rule out germ cell tumor, serum LDH helps to rule out lymphoma, TFT helps to rule out thyroiditis and serum anti-Acetyl choline antibodies help in diagnosing myasthenia gravis which is commonly associated with mediastinal thymoma (12). In our patient, none of these markers were elevated except TSH which was suggestive of hypothyroidism. 

Masaoka staging system is the most commonly used staging system for thymomas. Recently the tumor, node and metastasis (TNM) system of staging has also been incorporated for thymomas. However, all these staging systems cater to mediastinal lymphomas and there is no specific staging system for cervical thymomas. CECT remains the radiological investigation of choice and investigations like Positron Emission Tomography (PET) may not be useful in view of thymoma being a low grade tumor. CECT neck in our patient showed an extensive tumor that extended from the hyoid to D1 vertebra without any mediastinal extension. CECT chest showed multiple, variable-sized, subpleural and parenchymal nodules seen diffusely scattered in both the lungs. Based on these findings our patient was labelled as metastatic stage IV cervical thymoma and was considered unfit for surgical resection.

Upfront surgery with R0 resection is the primary treatment for thymomas. No adjuvant treatment is required if the capsule is intact and margins are clear in early stage I and II tumors (21-22). Higher Masaoka stage (III and IV) is associated with a greater probability of R1 resection (23). Safieddine et al and Forquer et al showed better outcomes with adjuvant radiation in all R1, R2 resections and stage III and IV tumors (24-25). Unresectable or medically inoperable disease may also be considered for definitive radiotherapy or multi-modality treatment consisting of induction chemotherapy, surgical resection, postoperative RT and consolidative chemotherapy (26). Radiation can be delivered by conformal radiation or intensity modulated radiation therapy to decrease toxicities in these patients with expected prolonged survival. Radiation doses in the range of 50-66 Gy can be used depending on the volumes being treated. 

Chemotherapy for thymomas includes the use of platinums, anthracyclines, taxanes, ifosfamide, etc with modest activity. 

National Comprehensive Cancer Network (NCCN) Panel recommends the cisplatin/doxorubicin/cyclophosphamide(CAP) regimen because it seems to yield the best outcomes. Non-anthracycline based regimens (cisplatin/etoposide) may be used in patients who cannot tolerate aggressive regimens (27-28). Therapy with Epidermal Growth Factor Receptor (EGFR) targeted agents or histone deacetylase inhibitors remains investigational. In our patient, we used a combination of CAP regimen. Unresectable disease can be treated with neoadjuvant chemotherapy for downstaging and improving resectability (23). In view of metastatic disease in our patient, she was planned for palliative chemotherapy with CAP regimen.

Prognostic factors for the mediastinal thymomas include stage, size of primary tumor, age at presentation and extent of resection. Tumors more than 10 cm in size have adverse prognosis while patients more than 30 years do better than younger patients. Complete surgical resection with clear margins is associated with favorable outcomes (29-33). Until standard guidelines are developed for ectopic thymomas; the staging, histopathological grading, prognostic factors, treatment regimens and outcomes of mediastinal thymomas will continue to be extrapolated to ectopic thymomas. Further studies may help us to better understand this rare disease.

## Conclusion

Thymoma is an uncommon tumor that typically does not present as an aggressive tumor. Ectopic thymic malignancy should be considered as a differential diagnosis during evaluation of a malignant neck mass. Histopathological and immunohistochemical confirmation would help in early detection, timely treatment and better outcomes. Cervical thymoma with lung metastases and without mediastinal involvement is a rare presentation and future studies on extra-mediastinal thymomas would help in better management of these rare tumors. 
